# The Epidemiology of Chemical Burns Among the Patients Referred to Burn Centers in Shiraz, Southern Iran, 2008–2018

**DOI:** 10.30476/BEAT.2021.90754.1261

**Published:** 2021-10

**Authors:** Hosein Abbasi, Ali Dehghani, Ali Akbar Mohammadi, Tayyeb Ghadimi, Abdolkhalegh Keshavarzi

**Affiliations:** 1 *Shiraz University of Medical Sciences, Shiraz, Iran *; 2 *Department of Nursing, Jahrom University of Medical Sciences, Jahrom, Iran *; 3 *Burn and Wound Healing Research Center, Shiraz University of Medical Sciences, Shiraz, Iran*; 4 *Burn Research Center, Iran University of Medical Sciences, Tehran, Iran *; 5 *Department of General Surgery, School of Medicine, Shiraz University of Medical Sciences, Shiraz, Iran*

**Keywords:** Chemical burn, Acid, Alkali, Epidemiology, Iran.

## Abstract

**Objective:**

To investigate the prevalence of chemical burns among the patients admitted to Shiraz burn treatment centers.

**Methods:**

It is a descriptive study that was conducted on 62 patients with chemical burns who were admitted between 2008 and 2018. The patients’ records were used in the research using the census sampling process. A questionnaire with questions about age, sex, the extent of the burn, the cause of the burn, duration of hospital stay, level of education, incident location, and clinical outcome was used to collect data (survival-death). The data was analyzed by using descriptive statistical methods.

**Results:**

The prevalence of chemical burns was 1% during 2008-2018. Acid and alkali burns were accounted for 93.5% and 6.5% of burns, respectively. 77.4% of patients were male, and 22.6% were female. The mean age of patients was 27 years. The average burn percentage was 16%. 70.6% of patients were illiterate or had primary education. Burns occurred at the workplace and home in 12.9% and 66.1% of cases, respectively. Moreover, Burns occurred due to accident (61%), acid attack (29%), and self-immolation (10%). The average length of hospital stay was 20 days. One patient (1.6%) died from burns.

**Conclusion:**

The study’s findings revealed that chemical burns were more common in men than women, and the majority of chemical burns occurred at home. To minimize the occurrence of chemical burns and acid attacks, teaching methods of preventing burns is important at home and work, as well as restricting non-specialists’ access to chemicals.

## Introduction

A burn is a tissue injury which can damage the skin, tissues, and underlying organs. Due to World Health Organization (WHO), in 2018, burns were identified as a public health problem worldwide, over 180,000 deaths and millions of injuries annually [1, 2]. The majority of burn accidents occur in developing and less developed countries, which are mainly due to the lack of attention to the principles of burn prevention, and lack of public training and awareness-raising [3], therefore, more than 95% of burn-related deaths occur in the middle- and low-income countries [4]. The burn is one of the most destructive forms of trauma and one of the most severe and risky events that affect the patients’ lives [4, 5]. On the other hand, burns impose huge economic costs to patients and their families, hospitals, medical centers and countries’ healthcare systems [4, 6].

One of the most important types of burns is chemical burns. A wide range of chemicals may cause skin and eye burns and systemic side effects through absorption or inhalation. Depending on the nature of the factors involved and the type of injuries, disabilities will be different. More than 25,000 chemicals are used in industry, agriculture, home, etc., many of which have the potential to cause burns [7, 8]. The causative agents of chemical burns may vary according to geographical location, population structure, and industrial structure. Acids and alkalis are the most common types of causative agents involved in chemical burns. For acids, sulfuric acid, hydrochloric acid and hydrofluoric acid are representative agents, and for alkalis, sodium hydroxide and potassium hydroxide are representative agents [9]. The variety of chemical agents is so vast that a short review cannot describe all the agents and their treatments, but we can provide general principles for the treatment of chemical injuries. The fact that they only represent near the 3% of all burns that must not underscore these principles. They are present with an important morbidity (near 55% of them require surgery), commonly involve cosmetic body like face, thorax and hands, and in some series they carry approximately 30% of burns death [10]. 

There is a great deal of disagreement regarding the epidemiology of chemical burns in different countries [11]. It is necessary to study this difference, which is due to epidemiological differences in the fields of population, culture, economy, the community among different countries and regions, to take preventive measures, comprehensive and effective laws to protect at-risk populations and thus to reduce the prevalence of chemical burns and subsequent complications. Due to the American Burn Association (ABA), chemical burn patients accounted for 3% of burn patients admitted to hospitals and burn centers during 2005-2014 [12].

Iran is a developing upper-middle income country with its own geographical, demographic, economic, and cultural characteristics, located in the Eastern Mediterranean Regional Office (WHO - EMRO), where the causes of burns vary over time due to economic, industrial, and social developments [13]. Therefore, despite the studies conducted on the epidemiology of burns in Iran, there has been no complete dimensions’ picture of this issue available for health and treatment decision-makers so far [14]. Although traffic accidents are the leading cause of mortality and morbidity in many countries worldwide, including Iran, burns are the 2^nd^ most common cause of death among children and the 3^rd^ most common cause of death due to incidents in all age groups in Iran. Burns are also among the 8^th^ main cause of death, the 13^th^ main cause of disability in Iran [15, 16].

There are few studies dedicated to the epidemiology and demographic information of chemical burns in Iran. There is only one epidemiology burns’ study caused by chemical and caustic substances in East Azerbaijan Chemical burn patients made up 2.4% of all burn patients admitted to the burn center of Sina Hospital in Tabriz during a 5-year period [17]. Other domestic studies that help investigate the epidemiology of chemical burns either investigate the epidemiology of only acid burn and acid attack cases or the epidemiology of burns related to all causes of burns in one province, medical center, one age group, or sex. In a study performed on patients with acid burns referred to Shahid Motahari Trauma and Burn Hospital in Tehran during 2006-2011, 171 acid-burn patients were reported, of which 24.6% were female, and 75.4% were male. Moreover, burn occurred due to accident and acid attacks in 88.9% and 11.1% of cases, respectively [18]. The importance of chemical burns includes its lasting effect, lack of basic information in province, the need to assessment long-term complications in the future and the development of similar research due to the impact of severe scars on mental condition of the injured. Therefore, this study aimed to investigate the epidemiology of chemical burns in Shiraz burn treatment centers over a 10-year period from 2008 to 2018.

## Materials and Methods

In this descriptive study, the study population were included patients admitted to Ghotbeddin and Amir Al-Momenin Burn Centers in Shiraz during 2008-2018. The medical records and records of hospitalized patients were reviewed due to the registry of the Burn and Wound Healing Research Center of the hospital from 2008-2018. The patients (5912) were referred to this center during this 10-year period, of which 62 patients had chemical burns, and therefore the same number was included. All patients admitted to the medical center were included in the study. The patients who were referred to the hospital for selective reasons, such as reconstructive surgery, were excluded from the study. Data collection was carried out by preparing a data collection form to register clinical information based on the registry. The studied variables included sex, age, burn percentage, cause of burn (accident, attack, self-immolation), the causative substance of burn, level of education, the geographical location of the residence place, accident location, marital status, length of hospital stay, and final condition of the patient (mortality rate). Data analysis was also carried out by using descriptive statistical methods, including frequency distribution tables and graphs, central tendency and dispersion indicators of study variables. A Chi-square test was used to investigate the relationship between sex and chemical agent. Mann–Whitney U test was also used to compare the mean burn percentage, mean age, and mean length of hospital stay between two groups of patients burnt with different chemical agents (acid and alkali).

The researchers kept confidential all registered medical information of patients archived in the medical records and burn patient’s data collection forms. This study results were published as whole and without mentioning the information and characteristics of patients.

## Results

The patients of 5912 were referred to Amir Al-Momenin, and Ghotbeddin Shirazi burn centers in Shiraz during 2008-2018, of which 62 suffering from chemical burns. They consisted of 58 acid-burn patients (93.5%) and 4 alkali-burn patients (6.5%). The burn pattern in our patients showed that flame and fire burns accounted for the highest frequency (44.7%) and chemical burns for the lowest frequency (1.0%). Other demographic characteristics are listed in Table 1.

**Table 1:** Frequency distribution of demographic characteristics of patients with chemical burns

**Percentage**	**Number**		**Variable**		
77.4	48		Male		Sex
22.6	14		Female		
93.5	58		Acid		Type of chemical substance
6.5	4		Alkali		
66.1	41		Home		Location of the accident
12.9	8		Workplace		
21	13		Outdoor		
29	18		Single		Marital status
67.8	42		Married		
3.2	2		Divorced		
27±19.2		Mean±SD		Age	
16.6±40.1		Mean±SD		Burn percentage	

**Fig. 1:**
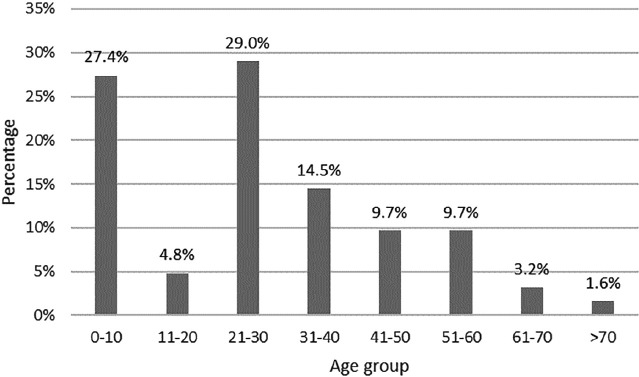
Frequency distribution of chemical burns in different age groups

The patients’ age range who referred to burn centers varied from 1.5-72 years. The mean age of patients was 27±19.2 years. The frequency distribution of chemical burns shows that the patients aged 21-30 years and under 10 years are considered high-risk groups, respectively (Figure 1). Also, patients aged 61 years and older are exposed to chemical burns less frequently (Figure 1).

The mean burn percentage was 16.6±11.4, therefore, burn percentage varied between 1-59% in the acid-burn group and 10-21% in the alkali-burn group. Mann-Whitney U test showed no significant difference between two groups due to mean burn percentage. Independent t-test showed no significant difference between men and women in terms of the average burn percentage (*p*=0.51) (Table 2).

**Table 2:** Comparison of the average burn percentage between two groups due to their sex

	**Gender**	**Number**	**Mean±SD**	**t**	**df**	***p*** ** value**
Burn percentage	Female	14	18.4±12.6	-0.66	60	0.51
	Male	48	16.1±11.1			

**Fig. 2.**
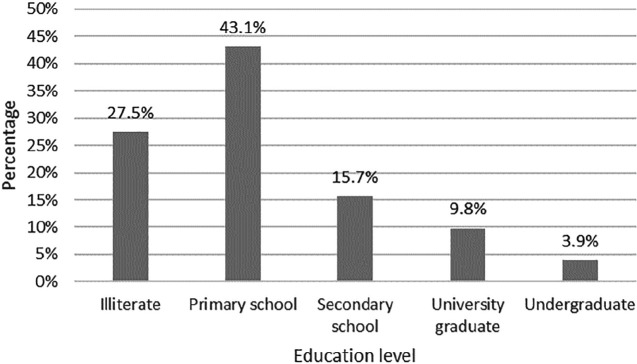
Education level of patients with chemical burn

Figure 2 shows the frequency distribution of burn patients with different education levels, and the highest burn rates were observed among the illiterate and undergraduates. The average length of hospital stay of patients with chemical burns was 20 days. Regarding alkali-burn patients, the average hospital stay is 2.5 days shorter than acid-burn patients. Moreover, the average length of hospital stay in women and men is 29 and 19 days, respectively. Mann-Whitney U test did not show a statistically significant difference between the two groups in terms of length of hospital stay by chemicals and sex. The findings also indicated that burns occurred in residential areas, workplace, and outdoors in 66%, 12.9%, and 21% of cases, respectively. Due to the results, out of 62 patients with chemical burns, only one died. The deceased patient was a 21-year-old married woman with burns=41% TBSA.

## Discussion

The present study also revealed that the average burn percentage was 16%. The prevalence of chemical burns was lower than most studies in other countries (1.4-8.5%) [15, 17-19]. In the American Burn Association (ABA), patients with chemical burns accounted for 3% of burn patients admitted to hospitals and burn centers during 2005-2014 [12]. Moreover, above association’s result showed that chemical burns had the lowest prevalence among the causes of burns, which is consistent to our study results. In a descriptive study on data from the burn registry in Australia and New Zealand, McInnes *et al*., [19] showed that chemical burns accounted for 5.8% of all burns among people aged 15-64 years. The higher prevalence of chemical burns reported by studies in the United States, Australia, and New Zealand than the results of our study shows that chemical burns account for a higher percentage of all burns in industrialized and developed countries, which seems to be due to the existence of larger industrial areas and the widespread use of chemicals in various industries [12, 19].

This study showed that men are more likely to work in at-risk environments. Men are also at greater risk of chemical burns due to exposure to chemicals or poor safety compliance. Ye *et al*., [20] found in a study in China that the ratio of chemical burns in men to women was 8.7: 1. Similarly, they showed in another study the above ratio was 3.6:1 [19, 20]. Maghsoudi and Jebraeili showed in a study in East Azerbaijan that the ratio of chemical burns in men to women was 10: 1 [17]. These findings are consistent to this study’s results. The above studies also indicate that a much higher prevalence of chemical burns in men is due to their greater employment in high-risk chemical and industrial occupations which is consistent to the much higher prevalence of chemical burns in industrial environments [18-20]. 

The caustic chemicals were the main cause of chemical burns. This study’s results are consistent to the results of another study showed acids were the cause of burns in 60% of cases [19], and a study by Ye *et al*., [20] where acids were the cause of burns in 72% of cases. The greater prevalence of acid burns may be due to the fact that acidic substances are more available depending on different uses. Another reason could be the false reduction of alkaline agents [21]. Acid chemical burns should be treated with immediate washing with copious amounts of water. For acid burns, Yano *et al*., [22] have shown that if washing was carried out within the first 3 min post injury (before the subcutaneous tissue pH level reached a minimum value), any remaining acid on the skin surface could be washed away, effectively suppressing the subsequent fall in PH. The experimental data by Yano *et al*., [22] were confirmed by previous clinical studies which demonstrated significantly less full skin thickness injury in patients who received immediate washing with copious amounts of water.

The highest burn percentage was observed in the 21-30-years age group (29%) and then children under 10 years of age (27%). Ye *et al*., [20] found that only 16% of the patients with chemical burns were under 30 years of age and the 40-49 and 30-39-year age groups had the highest frequency with 34% and 32%, respectively. There needs to be an increased community awareness regarding household chemicals especially amongst parents. The use of child-resistant locks on cabinets or doors where chemicals are stored, the practice of replacing them in a safe storage area after use, and the avoidance of transferring substances out of their original containers may reduce the risks to unattended children. Patel *et al*., [23] evaluated parental poison prevention practices and found that chemicals perceived by parents to be harmful were more likely to be stored properly, supporting the need for parental education. Finally, the poor use of first aid seen in both this study and that of Hardwicke *et al*., [24] would suggest that parents’ education and health professionals may be an effective tertiary prevention strategy.

Our study found that 12.9% of chemical burns occurred in the workplace, as a result of inappropriate machine operation, inappropriate chemical handling or machine problems. These data emphasize the importance of safety in the workplace and highlight the shortcomings of many enterprises in occupational education and training, machine maintenance, and production management.

Consistent to the study by Ye *et al*., [20] this study’s results showed that the highest prevalence of burns occurs in people with low educational levels, which necessitates the need for special burn prevention education for these people. Our survey showed that most patients had low educational attainment (primary school education), while only a small minority received college education or higher. This low level of education may partly explain the higher incidence of chemical burns in Fars province. It seems that taking more serious measures and requiring people, especially employees of various industrial centers, using protective equipment, as well as informing the general public about significant chemical damage can lead to a reduction in chemical injury.

One of the limitations of the present study is the small number of patients with chemical burns. Also, if the type of acid and alkali were determined in detail, more analysis would be carried out. Also, the inclusion of the injured body part (hands, feet, face, etc.) and degree of burn in the information record form could better express the details of the accident and clearer determination of the factors affecting the burn percentage and length of hospital stay.

This study’s findings showed that the prevalence of chemical burns was higher in men than women. The most of the burns occurred at home, which indicates that the importance of home-related chemical burns should also be taken into account in order to prevent the occurrence of chemical burns and in addition to making an effective decision to reduce occupational chemical burns. Moreover, the majority of chemical burns occurred among people aged less than 30 years of age and patients with low education. Therefore, it is recommended to provide the necessary training and care appropriate to children’s age and provide occupational and safety training, especially to adolescents and young people for reducing chemical burns.
